# Pregnancy Complications and Outcomes in Obese Women with Gestational Diabetes

**DOI:** 10.3390/medicina61010051

**Published:** 2025-01-01

**Authors:** Gitana Ramonienė, Laura Malakauskienė, Eglė Savukynė, Laima Maleckienė, Greta Gruzdaitė

**Affiliations:** Department of Obstetrics and Gynaecology, Medical Academy, Lithuanian University of Health Sciences, A. Mickevicius St. 7, 44307 Kaunas, Lithuania; gitana.ramoniene@lsmu.lt (G.R.); egle.savukyne@lsmu.lt (E.S.);

**Keywords:** gestational diabetes, obesity, pregnancy complications, labour complications

## Abstract

*Background and Objectives*: To assess pregnancy and delivery complications in obese women with gestational diabetes mellitus (GDM) and neonatal weight and condition after birth. *Materials and Methods*: A retrospective tertiary referral centre study included all cases of GDM in the Department of Obstetrics and Gynaecology of the Lithuanian University of Health Sciences (LUHS) Birth Registry from 1 January 2019 to 31 December 2019. We included 583 women with GDM and singleton pregnancies. Women were divided into two groups according to their pre-pregnancy weight: 202 were obese (BMI ≥ 30 kg/m^2^), and 381 were weight (BMI 18.5–24.9 kg/m^2^). Survey data were analysed using IBM SPSS Statistics 26.0 and MS Excel 2016 software. A value of *p* < 0.05 was considered significant. *Results*: Fasting glycaemia was significantly higher in obese women with GDM than in normal-weight women with GDM (*p* < 0.001). In addition, fasting glycaemia was higher in obese women with GDM requiring insulin correction than in normal-weight women (*p* = 0.006). OGTT 0-min glycaemia was higher in obese than in non-obese women with gestational diabetes (*p* < 0.001). Women with pre-pregnancy obesity had a higher incidence of primary hypertension (*p* < 0.001), hypertensive disorders in pregnancy (*p* < 0.001), gestational cholestasis (*p* = 0.002), polyhydramnios (*p* < 0.001), induced labour (*p* < 0.001), and caesarean section (*p* = 0.015), with emergency caesarean sections being more frequent than planned caesarean sections (*p* = 0.011) compared to normal-weight women with GDM. Labour dystocia (*p* = 0.003) and foetal distress (*p* = 0,019) were more common during labour in obese women. Neonates of these women more often had macrosomia (*p* < 0.001) and lower Apgar scores at 1 min (*p* = 0.024) and at 5 min (*p* = 0.024) compared to neonates of normal-weight women. *Conclusions*: Obese women with GDM experience more pregnancy complications than normal-weight women with GDM.

## 1. Introduction

Gestational diabetes (GD) is a disorder of carbohydrate metabolism characterised by hyperglycaemia of variable severity that is first diagnosed during pregnancy [1,2]. The screening of GD during pregnancy is performed between 24 and 28 weeks of gestation if it is not detected by fasting glycaemia at the beginning of pregnancy [3,4,5]. GD is one of the most common metabolic disorders detected in pregnancy [6]. Many reports indicated that the increasing number of patients with GD worldwide may be related to the growing epidemic of obesity [2,7,8].

The standard definition of obesity in the pregnant population is not easy to adapt because pregnant women gain weight in a relatively short period of time, and most of this is associated with weight gain that is lost during childbirth (consisting of the foetus, amniotic fluid, and blood). In the absence of a standard pregnancy-specific definition of obesity, pregnant women are classified as obese or non-obese based on their pre-pregnancy body mass index (BMI) [9].

Obesity is an important risk factor for the development of GD [2]. Obese pregnant women are at increased risk of various complications during pregnancy and labour. The risk increases with increasing body mass index [10]. Pregnancy in obese women diagnosed with GD is associated with worse pregnancy and delivery outcomes than either GD or obesity alone [11]. In addition, pre-pregnancy overweight and obesity are associated with an increased risk of pregnancy-induced hypertension, pre-eclampsia, an increased risk of emergency caesarean section (CS), and preterm birth [12].

Increased insulin resistance in obese pregnant women affects fetal metabolism: fetal blood glucose, ketones, free fatty acids and amino acids increase [13,14]. As a result, obese women are 1.4 to 1.8 times more likely to give birth to large-for-gestational-age newborns than women of normal weight [14]. Children born to obese women are also at higher risk of becoming obese in childhood and adulthood, as well as developing type 2 diabetes mellitus (DM) and cardiovascular disease [15].

Data from the “Health Behavior in the Lithuanian Adult Population” study showed that the rate of obese women of reproductive age is 24.1% [16]. We do not have data on the prevalence of obese pregnant women in our country. Obesity during pregnancy is a risk factor for complications in pregnancy and childbirth. Obesity and gestational diabetes are likely to have an even greater impact on complications for the women and the foetus. The aim of this study was to compare obstetric outcomes between obese and non-obese women with GDM delivering at a tertiary referral centre.

## 2. Materials and Methods

A retrospective study at a tertiary referral centre was conducted to review the obstetric records of women with GD who gave birth at 22 to 41 weeks of gestation at the Department of Obstetrics and Gynaecology, Lithuanian University of Health Sciences (LUHS) Hospital, from 1 January 2019 to 31 December 2019. The data were approved by the Kaunas Regional Bioethics Committee, Kaunas, Lithuania, under registration number BE-2-41. Data collected included maternal pre-pregnancy height and weight (BMI—weight in kilograms divided by height in metres squared (kg/m^2^) [17]), socio-demographic information (age, place of residence), number of previous pregnancies and births, duration of the current pregnancy, fasting venous plasma glucose (in the first trimester), and oral glucose tolerance test (OGTT) parameters at three glycaemic time points (0 min—fasting glycaemia, 60 min and 120 min glycaemia after ingestion of 75 g of glucose solution). This study also analysed the course of pregnancy and labour complications, including hypertensive disorders in pregnancy (pregnancy-induced hypertension, pre-eclampsia), anaemia, cholestasis, changes in the amount of amniotic fluid (polyhydramnios, oligohydramnios), methods of induction of labour, cephalopelvic disproportion (type of dystocia), other dystocia (prolonged labour related to the birth process), abnormal fetal position (breech presentation, transverse position), preterm birth (22 + 0 weeks to 36 + 6 weeks), mode of delivery (vaginal delivery or caesarean section, with indications for caesarean section analysed), bleeding due to uterine atony (data collected and analysed but not reported due to a small number of cases), chorioamnionitis, duration of labour and stage of delivery (in minutes), neonatal weight (in grams), assessed by sex and gestational age, and neonatal Apgar scores at 1 and 5 min.

The height and weight of the pregnant women were obtained from their medical records, and the first antenatal visit was made within the first 14 weeks of pregnancy. Obesity was diagnosed if the BMI was ≥30 kg/m^2^. The data collected in the study were compared between normal-weight women (18.5–24.9 kg/m^2^) and obese women with GD. Gestational diabetes is classified according to White: primarily diet-controlled GDM (class A1GDM) or GDM requiring pharmacological treatment (insulin therapy) of hyperglycaemia (class A2GDM) [18].

Hypertensive disorders in pregnancy are defined when arterial blood pressure (ABP) rises above 140/90 mmHg without proteinuria (pregnancy-induced hypertension) or with proteinuria (pre-eclampsia). Chronic (primary) hypertension and chronic (primary) hypertension complicated by pregnancy are diagnosed when elevated ABP and/or proteinuria are observed up to 20 weeks of gestation. Polyhydramnios is diagnosed in the presence of excess amniotic fluid as determined by ultrasound when the amniotic fluid index is greater than 240 mm or the deepest amniotic pocket is greater than 80 mm.

Foetal macrosomia was observed if the birth weight was greater than 4000 g. Excessive foetal or neonatal weight for gestational age is defined as greater than the 90th percentile for sex and gestational age.

The survey data were analysed using IBM SPSS Statistics 26.0 and MS Excel 2016 software. Descriptive data statistics—absolute (n) and percentage frequencies (%)—were used to assess the distribution of the analysed characteristics in the selected sample. The mean (m) and standard deviation (SD) used to describe the interval scale variables are given as the median (Me), minimum (min), and maximum (max) values of the variable. Distributions of two independent samples and unsatisfactory assumptions of normality were compared using the Mann–Whitney U test, while distributions of more than two independent samples were analysed using the Kruskal–Wallis test. The means of the quantitative variables of the two independent samples, for which the normality assumption was satisfied, were compared using the Student’s *t*-test. Tables of related variables, the chi-square (χ2) criterion for determining the homogeneity of variables, and the number of degrees of freedom (NDF) were calculated to assess the relationships between variables. Pairwise comparisons were performed using the z-test with Bonferroni correction. A value of *p* < 0.05 was considered significant.

We considered the relationship between variables to be statistically significant if the *p*-value was less than 0.05 (*p* < 0.05) and the statistical power of the 1-β test was 0.95 (1-β = 0.95). If these conditions are met, the effect size w = 0.25, and the number of degrees of freedom Df = 1–4 are chosen, the total sample size is 208–298. The survey data were analysed using IBM SPSS Statistics 26.0 and MS Excel 2016 software, G*Power V.3.1.9.4 University of Düsseldorf, Germany.

## 3. Results

According to data from the maternity register of the Department of Obstetrics and Gynaecology at LUHS Kauno klinikos, 3010 women gave birth at the maternity unit in 2019. Of our sample, 784 had gestational diabetes and gave birth between 22 and 41 weeks of gestation. In total, 583 women were selected for this study: 381 (65.3%) were of normal weight and 202 (34.7%) were obese. The BMI of 108 obese pregnant women was between 30 and 34.9 kg/m^2^ (53.5%), the BMI of 63 women was between 35 and 39.9 kg/m^2^ (31.2%), and the BMI of 31 women was ≥40 kg/m^2^ (15.3%).

Obese mothers were statistically significantly older than normal weight pregnant women (*p* = 0.012). Type A2 GD was significantly more common in obese women than in women of normal weight. Type A1 GD was diagnosed more often in normal-weight women than in obese women. Obese women were more likely to live in rural areas than normal-weight women. The median gestational age was 39 weeks in both groups and was not statistically significantly different (Table 1).

Obese women with gestational diabetes had a statistically significant increase in the number of pregnancies and births compared to non-obese women (*p* < 0.001). Obese pregnant women with type A2 GD were statistically significantly more likely to become pregnant and give birth three or more times than normal-weight women. The latter, normal-weight women, were more likely to give birth for the first time (Table 2).

In the first trimester of pregnancy, GD was detected in 224 women. Venous plasma glucose concentration was statistically significantly higher in obese women compared with normal-weight women. By type of gestational diabetes, fasting venous plasma glucose was statistically significantly higher in obese women with type A2 diabetes than in women of normal weight.

In the second and third trimesters of pregnancy, 359 women were diagnosed with gestational diabetes after OGTT. Fasting venous glucose (I OGTT point) was statistically significantly higher in obese than in normal-weight women. A significant difference was also found when comparing fasting glycaemia according to GD types A1 and A2 in obese and normal-weight women. The results of the second OGTT point (glycaemia 60 min after glucose ingestion) did not differ significantly. OGTT point III glycaemia (120 min after glucose ingestion) was significantly higher in normal-weight women with type A1 diabetes than in obese women with type A1 GD. The data are shown in Table 3.

Primary arterial hypertension was statistically significantly more common in obese pregnant women compared to normal-weight women (*p* < 0.001). Obese women with both GD A1 and GD A2 were statistically significantly more likely to develop primary arterial hypertension than normal-weight women. Hypertensive disorders in pregnancy (*p* < 0.001) and gestational cholestasis (*p* = 0.002) were diagnosed statistically significantly more often in obese pregnant women with GD than in those with normal body weight (type A1—*p* = 0.006, type A2—no significant difference; pre-eclampsia in obese women—6 (3.4%) and in normal-weight women—4 (3.2%)). The incidence of anaemia was not significantly different between the groups. The data are shown in Table 4.

**Figure 1:**
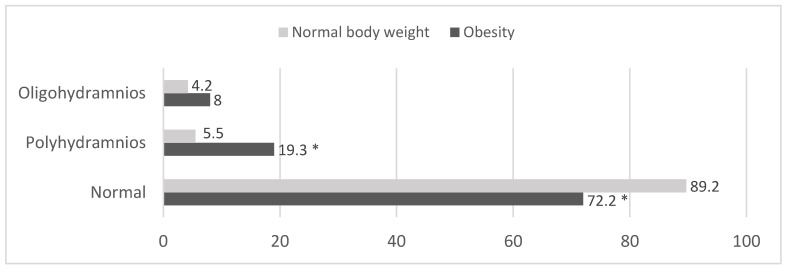
Distribution of normal-body-weight and obese pregnant women by amount of amniotic fluid (%) (N = 583). * *p* < 0.001 compared to women with normal body weight (z test with Bonferroni correction).

Normal amniotic fluid volume was statistically significantly more common in women of normal body weight than in women with GD (n = 340) and obese women (n = 146) (*p* < 0.001). Polyhydramnios was statistically significantly more frequent in the group of obese pregnant women (*p* < 0.001) (n = 39) compared to normal-weight women (n = 21) (Figure 1).

Comparing obese and normal-weight pregnant women by type of GD, both poly- and oligohydramnios were statistically significantly more common in obese pregnant women with type A1 GD compared to normal-weight women with type A1 GD (*p* = 0.003). Normal-weight women with type A2 gestational diabetes were statistically significantly more likely to have normal amniotic fluid levels compared to obese pregnant women with type A2 GD. In addition, in this group, polyhydramnios was statistically significantly more common in obese women compared to women with normal BMI. The data are shown in Table 5.

Labour was induced statistically significantly more often in obese women than in normal-weight women, regardless of the type of GD. Labour was induced in a total of 286 women. The most common method used was amniotomy, followed by medication (cervical ripening with misoprostol) and the least common by mechanical means (Foley catheter and other mechanical dilators). The analysis showed no statistically significant differences between the study groups regarding the choice of induction method. It is noteworthy that the number of cases of labour induction (177 in the normal weight group and 169 in the obese group) did not correspond to the number of births (151 in the normal weight group and 135 in the obese group) due to the use of multiple methods of labour induction for a single woman (Table 6).

Obese women with GD had a statistically significantly higher rate of caesarean section (*p* = 0.015) than in normal-weight women. Emergency caesarean section was also more frequent in the obese group (*p* = 0. 011). The data are shown in Table 7.

**Figure 2:**
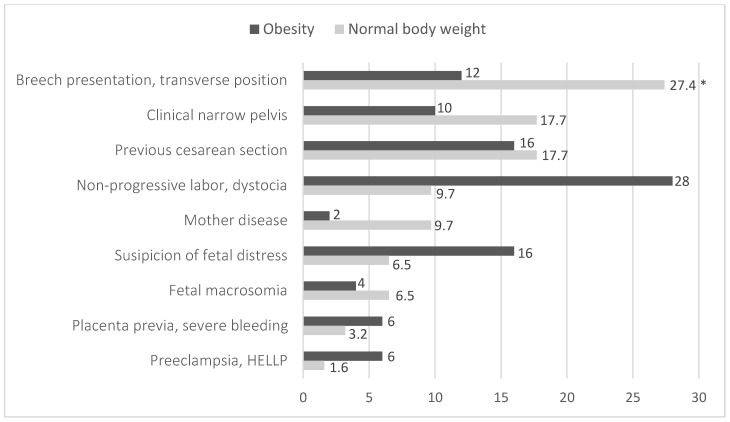
Distribution of normal-body-weight and obese pregnant women according to the causes for a C-section (N = 112). *p* = 0.032; *—*p* < 0.05, compared to women with normal body weight (z test with Bonferroni correction).

Caesarean section was performed statistically significantly more often in obese pregnant women with GD than in normal-weight women for suspected foetal distress—16% (n = 8), dystocia and non-progressive labour—28% (n = 14) compared to the frequency of these indications in normal-weight women—6.5% (n = 4) and 9.7% (n = 6), respectively (*p* = 0.032). Irregular position of the foetus in the uterus, i.e., breech presentation and transverse position, was statistically significantly more common as an indication for caesarean section in normal-weight pregnant women compared to the obese group (Figure 2).

The most common birth complications were chosen for the calculations. Only a few were statistically significant: foetal distress was more common in the obese group (*p* = 0.019) compared to the normal weight group. By type of GD, this complication was found to be statistically significantly more common in the A1 group of obese individuals. Dystocia was significantly more common in the obese group and in obese women with type A2 GD compared to normal weight mothers. Complications such as clinical pelvic obliquity, abnormal foetal position, chorioamnionitis and preterm delivery were similarly distributed in both groups with no statistically significant differences. The data are shown in Table 8.

The calculation and comparison of the median and mean duration of labour showed that the second stage of labour was statistically significantly longer in normal-weight women compared to obese pregnant women (Table 9).

No significant differences were found when comparing neonatal weights were compared without distinguishing between women by GD type, but neonates of normal-weight women with type A1 GD were statistically significantly heavier than those of obese women (*p* = 0.002). In contrast, in the A2 group, neonates of obese women were heavier than those of women of normal-weight women (*p* = 0.002). Apgar scores at 1 and 5 min were statistically significantly lower in neonates of obese women compared to neonates of normal-weight women. Foetal macrosomia was found to be statistically significantly more common in neonates in the obese women group than in neonates of normal-weight women. Obese pregnant women with both GD A1 and A2 were more likely to deliver macrosomic neonates than normal-weight women (Table 10). 

## 4. Discussion

This study aimed to evaluate maternal, foetal, and neonatal outcomes during pregnancy and delivery in obese and normal-weight women with gestational diabetes. This study showed that adverse pregnancy outcomes—foetal macrosomia, emergency caesarean delivery, and low post-delivery Apgar scores—were more common in obese pregnant women with GD than in normal-weight women with GD.

Obesity and gestational diabetes are conditions that significantly affect both the woman and the foetus before, during, and after pregnancy. It is, therefore, important to assess the pregnancy and birth complications associated with these metabolic disorders and to identify and treat them in time to minimise the consequences for the woman and the foetus.

In our study, a quarter of pregnant women with gestational diabetes were obese, just over a fifth were overweight, and almost half had a normal BMI. Similar data were obtained in a study by C. Machado et al., which included 3013 women diagnosed with GD, of whom 56.9% were overweight or obese [19]. In our study, gestational diabetes type A2 was diagnosed more often in obese women than in pregnant women with normal body weight, whereas GD type A1 was diagnosed more often in women who had a normal BMI before pregnancy. Based on these results, we can say that the carbohydrate metabolism of obese women is more altered, leading to a higher incidence of gestational diabetes requiring more intensive treatment, including diet, lifestyle changes, and insulin treatment. 

In our study, fasting plasma venous glucose in women diagnosed with GD in the first trimester of pregnancy was found to be statistically significantly higher in obese women than in women with normal pre-pregnancy weight. In addition, the first sample point (0 min, fasting) of the 2-hour 75 g OGTT was statistically significantly higher in obese pregnant women than in those with normal BMI diagnosed with GD in the second and third trimesters. Similar data were reported in the 2012 study by Baliutavičienė et al., who found that fasting glucose was higher in obese women (5.2 ± 1.1 mmol/L) than in normal-weight women (4.5 ± 0.8 mmol/L) [20]. It is important to note that in a recent study, gestational diabetes was diagnosed according to the guidelines for two glycaemic points. According to this OGTT methodology, GD was diagnosed but untreated if the fasting glycaemia was greater than 5.1 mmol/L. The same result is shown in a study by Sugiyama and co-authors, who found that the fasting point of the 2-hour 75 g OGTT was statistically significantly lower in the control group (normal-weight women) and higher in obese pregnant women. The second and third OGTT results were not statistically significantly different in our study; however, in the study by Sugiyama et al. study, both of these results were higher in the obese group compared to normal weight pregnant women [21]. Thus, these data again suggest that carbohydrate metabolism is altered obese women. Physiological changes in a woman’s body during pregnancy can affect metabolism, especially carbohydrate metabolism, leading to excessive glycaemia and an increased risk of complications during pregnancy and labour associated with hyperglycaemia.

In the studies by Sugiyama et al. and Baliutavičienė et al., as in our study, primary arterial hypertension was more common in obese pregnant women [20,21]. Hypertensive disorders in pregnancy, regardless of the type of GD, were statistically significantly more frequent in the obese group than in the normal weight group. The same data—hypertensive disorders in pregnancy statistically significantly more common in pregnant women—are published in many other studies describing the complications of pregnancy and labour in obese pregnant women with GD [20,21,22,23].

Polyhydramnios was more frequent in the group of obese pregnant women than in the group of normal-weight women. Similar data were found in the study by Baliutavičienė et al. [20].

Obese women were statistically significantly more likely to have induced labour than women with a normal BMI, and their second stage of labour was shorter than that of women of normal weight. This may be due to higher birth rates and epidural analgesia, but we did not analyse these associations. Similar data were reported in other studies showing that obese women were more likely to have induced labour than a group of normal-weight women [21,24]. Our study found that obese pregnant women were more likely to deliver by CS than normal-weight mothers. The same data are described in the studies by Lithuanian and foreign authors [11,19,20,22,23]. In many studies, obesity and gestational diabetes are independent risk factors for CS, but both may increase the risk of foetal macrosomia and influence the higher number of CS [19]. According to our study, dystocia as a complication of labour was more frequent in obese pregnant women. The largest sample (n = 1,057,647) in the study by Whiteman et al. describes that there was no statistically significant difference in the incidence of dystocia between obese and normal weight pregnant women [11]. The difference in results may be due to the different types of dystocia included in the study data.

One of the most commonly described complications of obesity, especially gestational diabetes, is overweight foetuses. In our study, neonatal birth weight was not statistically significantly different between groups, but macrosomia was several times more common in the obese group than in the normal weight group. Authors in many other countries have also found that macrosomia is more common in obese mothers with GD than in normal-weight women with gestational diabetes [6,17,22,25,26,27].

There are several limitations to this study. The most important is the relatively small sample size of this research. Because of this limitation, amniotic fluid volume (polyhydramnios and oligohydramnios) in women with foetal macrosomy and foetal hypotophy is not compared.

Due to the small sample size, we were not able to analyse the data by obesity class. The next planned direction of research is to assess the complications of pregnancy and childbirth by obesity class.

## 5. Conclusions

Pregnancy complications were significantly more common in obese pregnant women with gestational diabetes. Obese pregnant women with gestational diabetes had higher glucose levels and were more likely to be diagnosed with primary and hypertensive disorders in pregnancy, cholestasis, polyhydramnios, and foetal macrosomia. They were also more likely to have an emergency caesarean section at birth, to be diagnosed with labour dystocia, and to have lower Apgar scores than normal-weight women with gestational diabetes.

## Figures and Tables

**Table 1:** Socio-demographic factor (N = 583).

Criteria		Women of Normal Weight	Obese Women	*p*-Value
		n = 381 (%)	n = 202 (%)	
Age (years) ^1^		31.04 ± 5.4	32.22 ± 5.3	*p* = 0.012
Type of GD ^2^	A1	271 (71.1)	104 (51.4)	*p* < 0.05
	A2	110 (28.9)	98 (48.5)	
Place of residence ^2^	City	270 (70.9)	126 (62.4)	*p* = 0.041
	Countryside	111 (29.1)	76 (37.6)	
Weeks of gestation ^3^		39 (24–41; 38.63)	39 (22–41; 38.47)	*p* = 0.140

^1^—data presented as mean (standard deviation, SD (±), *p*-value—Student’s *t*-test; ^2^—n (%), *p*-value—*x*^2^ the homogeneity of variables criteria; ^3^—data presented as Me (min-max; mean), *p*-value—Mann–Whitney U test.

**Table 2:** The number of pregnancies and deliveries (N = 583).

Criteria			Women of Normal Weight	Obese Women	*p*-Value
			n = 381(%)	n = 202 (%)	
The number of pregnancies ^2^			2 (1–6; 1.98)	2 (1–9; 2.58)	*p* < 0.001
The number of pregnancies according to type of GD ^1^	A1	First	122 (45.0)	39 (37.5)	*p* = 0.150
		Second	86 (32.0)	31 (29.8)	
		Third or more	63 (23.0)	34 (32.7)	
	A2	First	39 (35.5)	17 (17.3)	*p* = 0.009
		Second	35 (31.8)	34 (34.7)	
		Third or more	36 (32.7)	47 (48.0)	
The number of deliveries ^2^			2 (1–6; 1.72)	2 (1–9; 2.06)	*p* < 0.001
The number of deliveries according to type of GD ^2^	A1	First	140 (51.7)	55 (52.9)	*p* = 0.303
		Second	95 (35.0)	30 (28.8)	
		Third or more	36 (13.3)	19 (18.3)	
	A2	First	49 (44.5)	21 (21.4)	*p* = 0.002
		Second	38 (34.5)	47 (48.0)	
		Third or more	23 (20.9)	30 (30.6)	

^1^—n (%), *p*-value—*x*^2^ the homogeneity of variables criteria; ^2^—data presented as Me (min-max; mean), *p*-value—Mann–Whitney U test.

**Table 3:** Comparison of mean venous plasma glucose level and mean venous plasma glucose level at exact OGTT points in normal-weight vs. obese pregnant women with GD (isolating type A1 and A2 according to White) diagnosed in the first third of pregnancy (N = 583).

Test		Normal Body Weight n = 381 (%)	Obesity n = 202 (%)	*p*-Value
Venous plasma glucose level (mmol/L), (n = 224)		5.49 (5–8; 5.56)	5.67 (5–7; 5.78)	*p* < 0.001
Venous plasma glucose level according to type of GD (mmol/L)	A1	5.49 (5–8; 5.56)	5.62 (5–7; 5.71)	*p* = 0.106
	A2	5.48 (5–7; 5.58)	5.69 (5–7; 5.86)	*p* = 0.006
OGTT I point (0 min) (mmol/L), (n = 359)		5.20 (3–8; 5.19)	5.45 (3–8; 5.52)	*p* < 0.001
OGTT I point (0 min) according to type of GD (mmol/L)	A1	5.20 (3–8; 5.09)	5.42 (3–7; 5.40)	*p* < 0.001
	A2	5.26 (4–8; 5.41)	5.52 (4–8; 5.58)	*p* = 0.025
OGTT II point (60 min) (mmol/L), (n = 359)		8.10 (4–14; 8.28)	8.29 (3–13; 8.48)	*p* = 0.105
OGTT II point (0 min) according to type of GD (mmol/L)	A1	8.00 (4–14; 8.25)	7.80 (3–13; 8.02)	*p* = 0.550
	A2	8.15 (4–14; 8.38)	8.75 (5–13; 8.97)	*p* = 0.071
OGTT III point (120 min), (mmol/L), (n = 359)		7.40 (4–15; 7.56)	7.39 (3–11; 7.33)	*p* = 0.453
OGTT III point (0 min) according to type of GD (mmol/L)	A1	7.56 (4–15; 7.77)	7.10 (3–10; 7.08)	*p* = 0.037
	A2	7.10 (4–13; 7.10)	7.64 (5–11; 7.58)	*p* = 0.104

The data in the table is presented as Me (min-max; mean), *p*-value—Mann–Whitney U test.

**Table 4:** Distribution of pregnant obese and normal-body-weight women with GD (isolating A1 and A2 types according to White) according to pregnancy complications (N = 299).

Pregnancy Complication		Normal Body Weight n = 126 (%)	Obesity n = 173 (%)	*p*-Value
Primary hypertension		11 (2.9)	38 (18.8)	*p* < 0.001
Primary hypertension according to type of GD	A1	6 (2.2)	15 (14.4)	*p* < 0.001
	A2	5 (4.5)	23 (23.5)	*p* < 0.001
Hypertensive disorders in pregnancy		72 (19.0)	96 (47.5)	*p* < 0.001
Hypertensive disorders in pregnancy according to type of GD	A1	51 (19.0)	44 (42.3)	*p* < 0.001
	A2	21 (19.1)	52 (53.1)	*p* < 0.001
Gestational anemia		37 (9.8)	26 (12.9)	*p* = 0.251
Gestational anemia according to type of GD	A1	24 (8.9)	12 (11.5)	*p* = 0.443
	A2	13 (11.8)	14 (14.3)	*p* = 0.597
Cholestasis of pregnancy		6 (1.6)	13 (6.4)	*p* = 0.002
Cholestasis of pregnancy according to type of GD	A1	5 (1.9)	8 (7.7)	*p* = 0.006
	A2	1 (0.9)	5 (5.1)	*p* = 0.071

Data presented n (%), *p*-value—*x*^2^ the homogeneity of variables criteria.

**Table 5:** Distribution of normal-body-weight and obese pregnant women with A1 and A2 types of gestational diabetes by amount of amniotic fluid (N = 583).

Type of GD	Amount of Amniotic Fluid	Normal Body Weight n = 381, n (%)	Obesity n = 202, n (%)	Statistics
A1	Normal	240 (88.5)	77 (74.0)	*p* = 0.003
	Polyhydramnios	17 (6.3)	16 (15.4) ^1^	
	Oligohydramnios	12 (4.5)	11 (10.6) ^1^	
A2	Normal	102 (92.7)	69 (23.7) ^2^	*p* < 0.001
	Polyhydramnios	4 (3.6)	23 (23.7) ^2^	
	Oligohydramnios	4 (3.6)	5 (5.2)	

^1^—*p* < 0.05, compared to normal-body-weight women with type A1 gestational diabetes; ^2^—*p* < 0.05, compared to normal-body-weight women with type A1 gestational diabetes (z test with Bonferroni correction).

**Table 6:** Labour of normal-body-weight and obese women with GD (A1 and A2 types). (N = 519).

Criteria			Normal Body Weight n = 340(%)	Obesity n = 179(%)	*p*-Value
Spontaneous onset of labour			189 (55.6)	44 (24.6)	*p* < 0.001
Induced labour	Amniotomy Mechanical methods Using drugs		126 (83.4) 9(6.0) 42 (27.8)	111 (82.2) 13 (9.7) 45 (33.3)	*p* > 0.001
Labour according to the type of GD	A1	Spontaneous	143 (59.1)	27 (29.7)	*p* < 0.001
		Induced	99 (40.9)	64 (70.3)	
	A2	Spontaneous	46 (46.9)	17 (19.3)	*p* < 0.001
		Induced	52 (53.1)	71 (80.7)	

The data in the table is presented n (%), *p*-value—*x*^2^ the homogeneity of variables criteria.

**Table 7:** Type of delivery of normal-body-weight and obese pregnant women.

Criteria		Normal Body Weight n (%)	Obesity n (%)	*p*-Value
Type of delivery (n = 583)	Vaginal	319 (83.5)	152 (75.2)	*p* = 0.015
	CS	62 (16.5)	50 (24.8)	
Urgency of CS (n = 112)	Planned	36 (58.1)	17 (34.0)	*p* = 0.011
	Emergency	26 (41.9)	33 (66.0)	

The data in the table is presented n (%), *p*-value—*x*^2^ the homogeneity of variables criteria.

**Table 8:** Distribution of pregnant obese and normal-body-weight women with GD (isolating A1 and A2 types according to White) according to delivery complications (N = 583).

Complications of Delivery		Normal Body Weight n = 151 (%)	Obesity n = 135 (%)	*p*-Value
Clinical narrow pelvis		9 (2.4)	4 (2.0)	*p* = 0.759
Distribution of clinical narrow pelvis according to the type of GD	A1	7 (2.6)	3 (2.9)	*p* = 0.880
	A2	2 (1.8)	1 (1.0)	*p* = 0.630
Foetal distress		4 (1.1)	8 (4.0)	*p* = 0.019
Distribution of foetal distress according to the type of GD	A1	2 (0.7)	5 (4.8)	*p* = 0.009
	A2	2 (1.8)	3 (3.1)	*p* = 0.559
Dystocia		13 (3.4)	19 (9.4)	*p* = 0.003
Distribution of dystocia according to the type of GD	A1	10 (3.7)	7 (6.7)	*p* = 0.211
	A2	3 (2.7)	12 (12.2)	*p* = 0.008
Abnormal foetus position		17 (4.5)	6 (3.0)	*p* = 0.372
Distribution of abnormal foetus position according to the type of GD	A1	14 (5.2)	3 (2.9)	*p* = 0.335
	A2	3 (2.7)	3 (3.1)	*p* = 0.886
Chorioamnionitis		16 (4.2)	8 (4.0)	*p* = 0.880
Distribution of chorioamnionitis according to the type of GD	A1	12 (4.5)	7 (6.7)	*p* = 0.371
	A2	4 (3.6)	1 (1.0)	*p* = 0.219
Preterm delivery		27 (7.1)	14 (6.9)	*p* = 0.931
Distribution of preterm delivery according to the type of GD	A1	20 (7.4)	10 (9.6)	*p* = 0.487
	A2	7 (6.4)	4 (4.1)	*p* = 0.463

The data in the table is presented n (%), *p*-value—*x*^2^ the homogeneity of variables criteria.

**Table 9:** Comparison labour duration in normal-body-weight and obese pregnant women with A1 and A2 gestational diabetes (m ± SN).

Duration of Delivery		Normal Body Weight n = 381	Obesity n = 202	*p*-Value
Total		500 (10–1764; 582.76)	420 (69–1850; 558.80)	*p* = 0.495
Total, according to the type of GD	A1	493 (60–1764; 595.41)	524 (69–1850; 644.80)	*p* = 0.343
	A2	525 (10–1465; 551.78)	342 (81–1610; 466.58)	*p* = 0.094
I stage of labour		430 (4–1740; 502.45)	360 (5–1590; 466.6)	*p* = 0.279
I stage of labour according to the type of GD	A1	425 (25–1740; 513.65)	465 (5–1590; 559.63)	*p* = 0.323
	A2	450 (4–1320; 475.12)	310 (40–1530; 367.55)	*p* = 0.017
II stage of labour		30 (24–310; 61.89)	24 (2–310; 47.70)	*p* = 0.030
II stage of labour according to the type of GD	A1	31 (1–314; 62.14)	30 (2–268; 54.74)	*p* = 0.378
	A2	27 (1–288; 57.82)	18 (2–310; 39.79)	*p* = 0.070

The data in the table is presented as Me (min-max; mean), *p*-value Mann–Whitney U test.

**Table 10:** Comparison of the data of neonates of normal-body-weight and obese pregnant women (N = 583).

Criteria		Normal Body Weight	Obesity	*p*-Value
Mean of neonate weight (g±SD), (n = 583) ^1^		3343 ± 617	3444 ± 694	*p* = 0.074
Distribution of the mean of neonates weight according to the type of mother’s GD, (g ± SD) ^1^	A1	3337 ± 648	3314 ± 827	*p* = 0.002
	A2	3357 ± 537	3581 ± 484	*p* = 0.002
Mean of Apgar scores after 1 min., (n =583) ^2^		9 (3–10; 9.06)	9 (2–10; 8.9)	*p* = 0.024
Distribution of the mean of Apgar scores after 1 min according to the type of mother’s GD, (scores±SD) ^2^	A1	9 (4–10; 8.99)	9 (2–10; 8.66)	*p* = 0.027
	A2	9 (3–10; 9.23)	9 (5–10; 9.15)	*p* = 0.537
Mean of Apgar scores after 5 min., (n = 583) ^2^		10 (6–10; 9.66)	10 (6–10; 9.52)	*p* = 0.024
Distribution of the mean of Apgar scores after 5 min according to the type of mother’s GD ^2^	A1	10 (6–10; 9.65)	10 (6–10; 9.38)	*p* = 0.537
	A2	10 (8–10; 9.7)	10 (8–10; 9.67)	*p* = 0.725
Macrosomia, (n = 84) ^3^		38 (10.0)	46 (22.8)	*p* < 0.001
Distribution of macrosomia according to the type of mother’s GD ^3^	A1	28 (10.4)	23 (22.1)	*p* = 0.003
	A2	10 (9.1)	23 (23.5)	*p* = 0.005
Foetal growth restriction (n = 83) ^3^		51 (13.5)	32 (15.8)	*p* = 0.434

^1^—data presented as mean (standard deviation, SD (±), *p*-value—Student’s *t*-test; ^2^—data presented as Me (min–max; mean), *p*-value—Mann–Whitney U test; ^3^—n (%), *p*-value—*x*^2^ the homogeneity of variables criteria.

## Data Availability

Anonymised data used for the study will be stored on a separate biomedical study data storage computer that was used to conduct the biomedical study. The data will be kept for 15 years after the study and then destroyed.
